# Supplementary material for: Dynamic movement of the Golgi unit and its glycosylation enzyme zones
Source: Nat Commun. 2024 May 27;15:4514. doi: 10.1038/s41467-024-48901-1 (PMC11130159; doi:10.1038/s41467-024-48901-1)
Supplement: Supplementary file 1 — Supplementary Information [file 41467_2024_48901_MOESM1_ESM.pdf]

Supplementary Figure 1

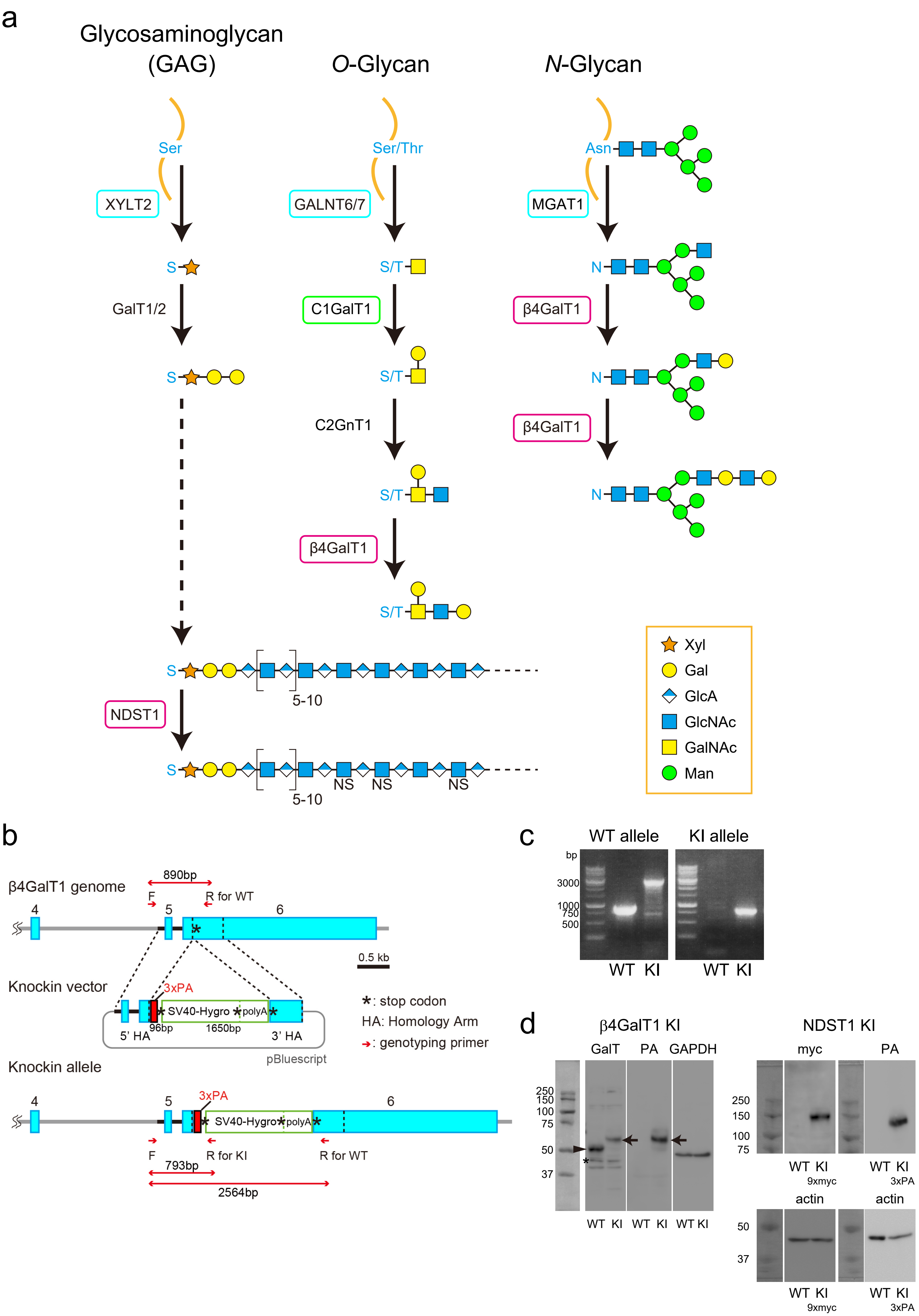

Supplementary Figure 1

Schematic view of three glycosylation pathways for proteins and validation of tag knockin.

a Enzymes appearing in this paper are shown in blue and red rectangles for early- and late-step enzymes, respectively. C1GalT1, an enzyme at the next step after GALNT6/7, is shown in a green rectangle.

b Schematic representation of knockin of tags into the last coding exon of β4GalT1.
The positions of primers for genotyping of WT and knockin (KI) alleles are shown as red arrows.

c Genotyping of a β4GalT1-3xPA KI clone which is homozygous for the KI allele (KI/KI).
Left, PCR product using primer pairs to detect the wild-type allele. In the wild-type clone, an 800-900 bp band was detected. In the KI clone, only a 2-3 kb long band is detected due to the insertion of the 3xPA tag and drug resistance gene in the exon, indicating that the wild-type allele is absent in this KI clone.
Right, PCR product using primer pairs to detect the KI allele.

d Western blot of wild-type and KI clones.
Left, Western blot for β4GalT1-3xPA KI stained with anti-β4GalT1 (left two lanes), anti-PA (middle two lanes), and anti-GAPDH (right two lanes) antibody. An asterisk indicates a nonspecific band.
Right, (top) Western blot for NDST1 KI clones. The NDST1-9xMyc (left two lanes) KI clone was stained with anti-Myc antisera, and the NDST1-3xPA (right two lanes) KI clone was stained with anti-PA antibody to confirm that their molecular weight was similar to the calculated molecular weight.
(bottom) Western blot for actin as a loading control.

Supplementary Figure 2

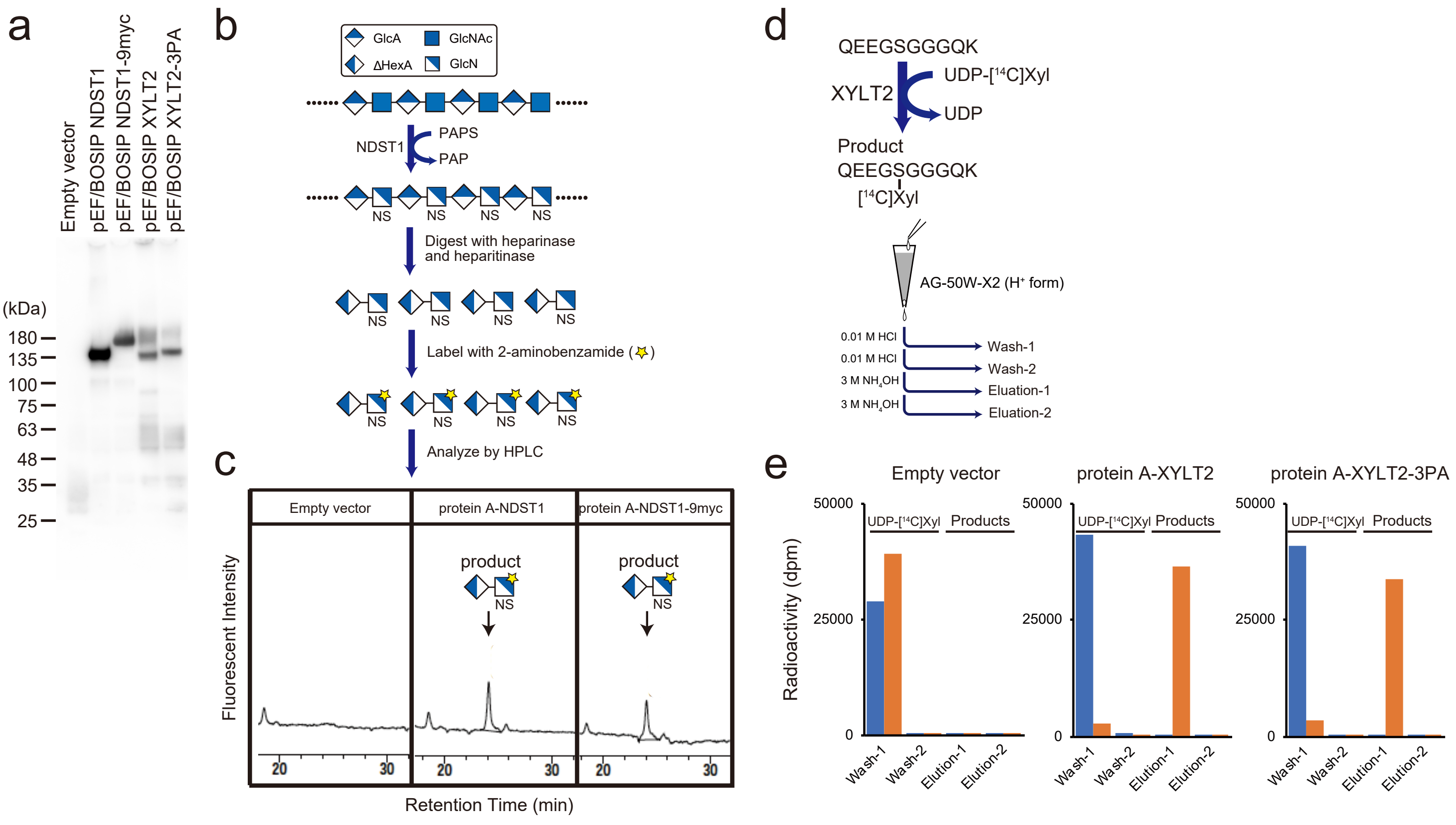

Supplementary Figure 2

Enzyme activity of 9xMyc- and 3xPA-tag attached to NDST1 and XYLT2.

a Protein A-NDST1, protein A-NDST1-9xMyc, protein A-XYLT2, and protein A-XYLT2-3xPA expressed in COS7 cells were collected via IgG-Sepharose and analysed by immunoblotting.

b Schematic illustration of the enzyme assay for NDST1. Expressed NDST1 catalyses the sulfation of K5 polysaccharides (non-sulfated heparosan).

Sulfated K5 polysaccharides were digested with a mixture of heparitinase and heparinase.

The resulting disaccharides were analysed by HPLC.

c NDST1-reaction products were digested with a mixture of heparitinase and heparinase and then analysed by HPLC. Arrows indicate the elution position of the product generated by the action of NDST1.

d Schematic illustration of the enzyme assay for XYLT2. XYLT2 catalyses the transfer of [14C]-labelled xylose to the inter-a-trypsin inhibitor-derived peptides. Reaction products were separated by a chip column as described in Methods section.

e The radioactivity of Wash-1, Wash-2, Elution-1, and Elution-2 fractions recovered from a chip column was measured by liquid scintillation counting.

Supplementary Figure 3

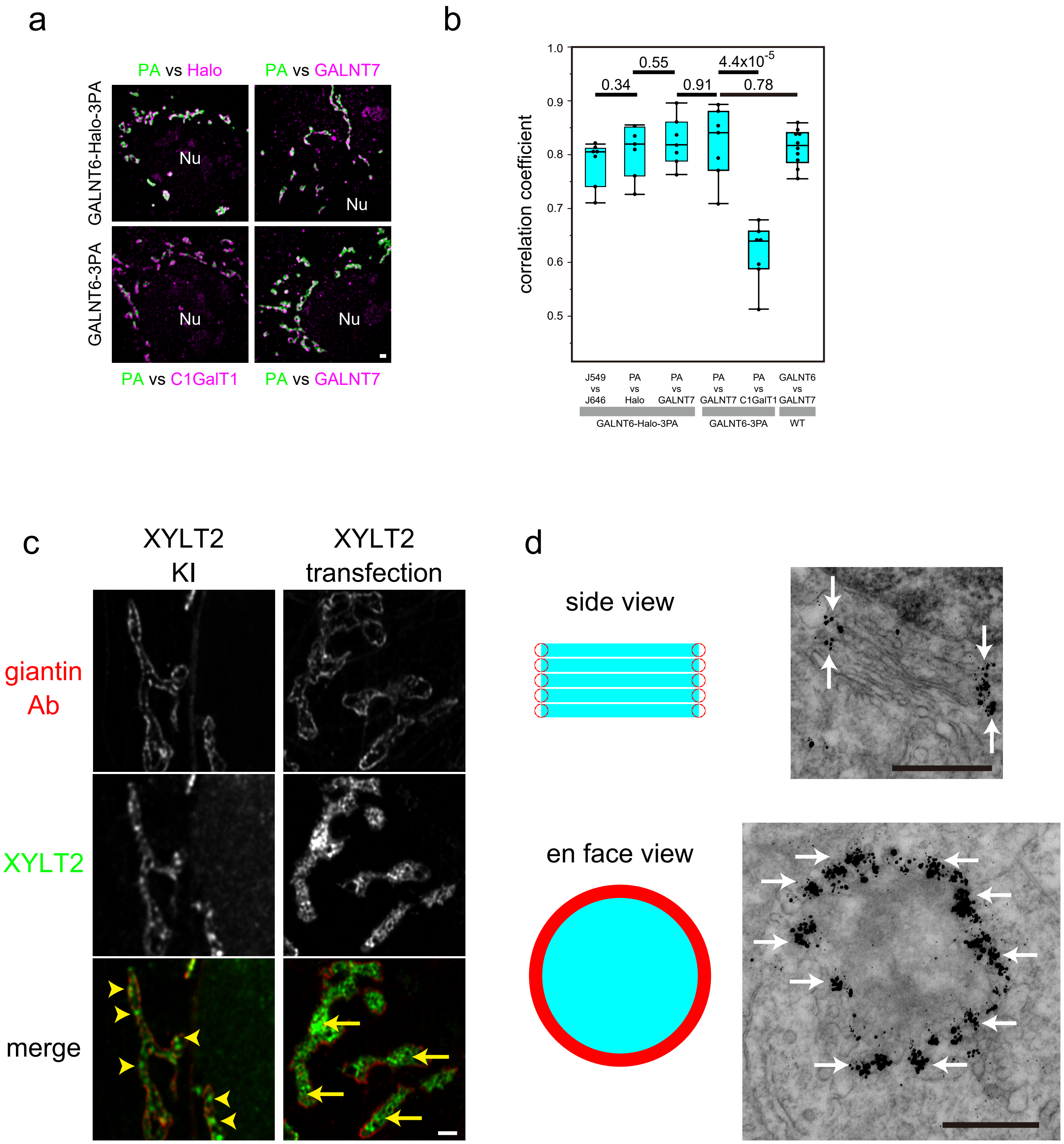

Supplementary Figure 3

Validation of knockin and localisation of giantin

a Two-colour SCLIM 3D imaging of KI cells stained with antibodies except the left upper cell, which is double stained with anti-PA antibody and a J549-tagged Halo substrate.

Upper panels: GALNT6-Halo-3xPA KI cells. Lower panels: GALNT6-3xPA KI cells. Nu: nucleus. Bar, 1 µm.

b 3D colocalisation analysis among cells in a.

In GALNT6-Halo-3xPA knockin (KI) cells, the correlation coefficient between different Halo substrates (J549 and J646) and between PA- and Halo-tags was approximately 0.8, indicating that a correlation coefficient of 0.8 corresponds to complete colocalisation.

We used an antisera against another GALNT, GALNT7, to evaluate the localisation of different GALNTs. The correlation coefficient between GALNT6 and GALNT7 was close to 0.8, indicating that both almost completely colocalised. The result also indicates that GALNT7 can be utilized to determine whether the localisation of tagged GALNT6 protein preserves its endogenous localisation.

When we compared the localisation of PA tags in GALNT6-Halo-3xPA and GALNT6-3xPA KI cells with GALNT7, their correlation coefficients were not significantly different from that between GALNT6 and 7. Therefore, the 3xPA tag and a Halo tag do not exert a significant effect on the localisation of GALNT6.

The correlation coefficient between C1GalT1, an O-glycosylation enzyme that catalyses the step after GALNTs, and GALNT6 was significantly lower than 0.8, suggesting that C1GalT1 and GALNT6 localise at distinct places.

Each point represents the value from one cell. Thus, the number of cells is more than 6.

Statistical significance was determined by two-tailed unpaired t test. P values are depicted in the graph.

c Comparison of the localisation of XYLT2 by Airyscan microscopy.

Left, Immunofluorescence of an XYLT-3PA KI cell.

Right: Immunofluorescence of a cell transfected with XYLT2-flag.

Arrowheads indicate punctate staining of XYLT2 in KI clones. Arrows indicate diffuse staining of XYLT2 by transfection. Bar, 1 µm.

d Localisation of giantin by immunoelectron microscopy.

Left, Expected localisation of giantin (red) in the side view (top) and the en face view (bottom).

Right, immunoelectron micrographs showing the localisation of giantin (arrows) in the side view (top) and the en face view (bottom). Bars, 500 nm.

Supplementary Figure 4

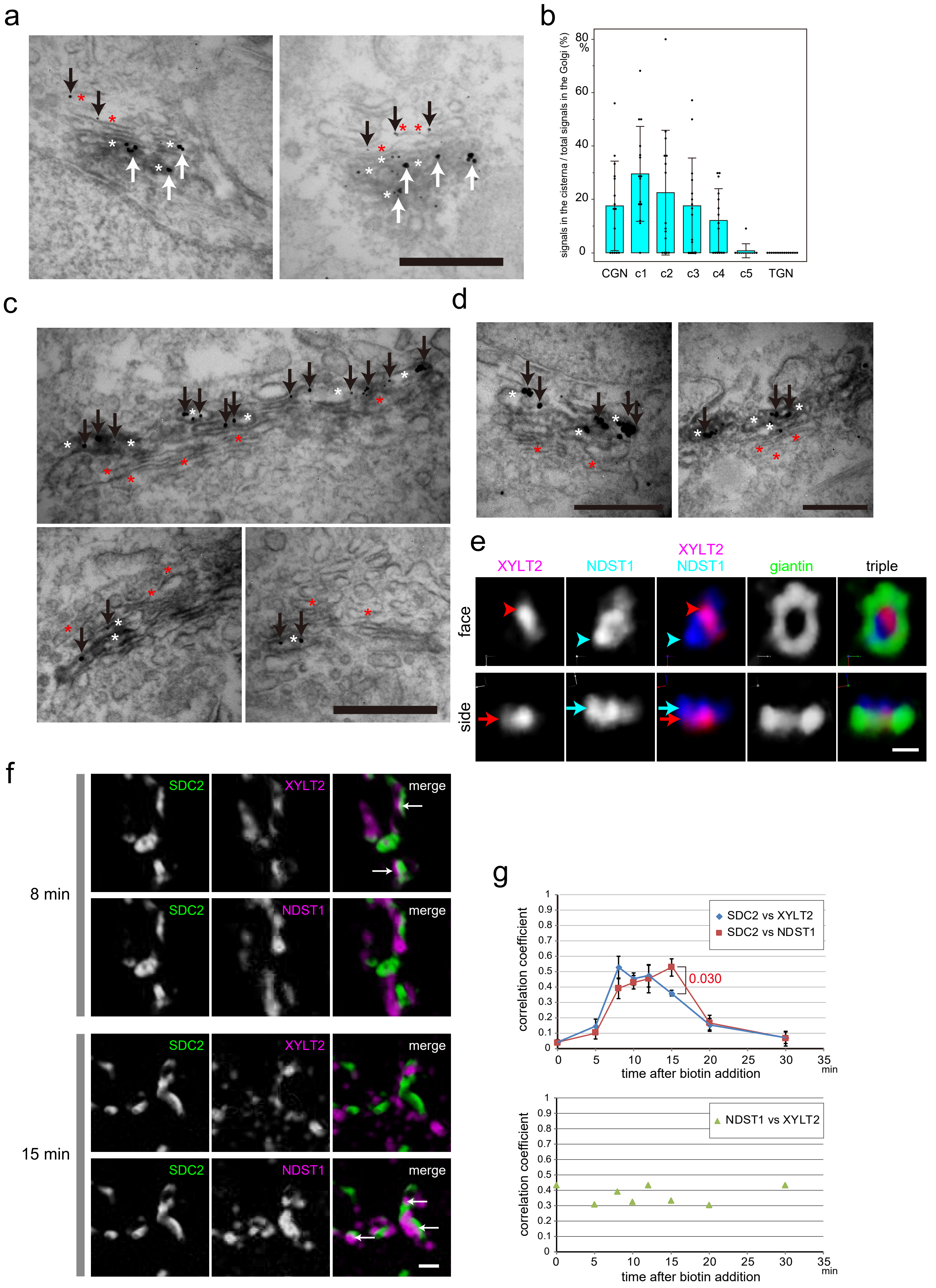

Supplementary Figure 4

Localisation of glycosylation enzymes revealed by immunoelectron microscopy.

a Double immunoelectron micrographs showing GALNT6 and GM130 in GALNT6-9xMyc KI cells.
Endogenous GM130 was stained with DAB. GM130-positive dark cisternae are indicated by white asterisks. GM130-negative cisternae are indicated by red asterisks. GALNT6 was labelled by nanogold followed by silver enhancement. GALNT6 signals are distributed mainly in GM130-positive cisternae (white arrows).
However, a small fraction of GALNT6 is also localised in GM130-negative cisternae (black arrows). Bar, 500 nm.

b A bar graph showing the distribution of GALNT6 in the Golgi stacks.
The percentage of signals localised in each cisterna was measured in a single Golgi complex. N=11.
As most of the Golgi contain five cisternae and adjacent cis- and trans-networks, we used the Golgi, which exhibits this configuration. The percentage of the vertical axis shows the percentage of # of dots (signals) in the cisternae/# of all dots (signals) in the Golgi. The height of the bars represents the average, and the error bars represent the SD.

c Immunoelectron micrographs of three cells showing GALNT6 and XYLT2 in GALNT6-9x Myc and XYLT2-3xPA double KI cells.
XYLT2 is stained as dots (arrows), which are localised in darkly stained GALNT6-positive Golgi cisternae (white asterisks) but not in lightly stained GALNT6-negative cisternae (red asterisks).
By this method, GALNT6 was detected by the HRP-labelled secondary antibody, and the resultant DAB reaction products diffused throughout the cisternae. Therefore, it was difficult to precisely compare the localisation of these two enzymes within a cisterna by this method. Bar, 500 nm.

d Double immunoelectron micrographs showing NDST1 and β4GalT1 in an NDST1-9xMyc KI cell.
Endogenous β4GalT1 was stained with DAB. β4GalT1-positive dark cisternae are indicated by white asterisks. β4GalT1-negative cisternae are indicated by red asterisks. NDST1 was labelled by nanogold followed by silver enhancement. NDST1 signals are mostly distributed in β4GalT1-positive cisternae (white asterisks). Bar, 500 nm.

e Three-colour SCLIM 3D imaging of a Golgi unit in XYLT2-3xPA and NDST1-9xMyc KI cells stained with antibodies against Myc and PA tags in the en face view (top) and the side view (bottom).
XYLT2 and NDST1 do not extensively overlap in the en face view. Bars, 1 µm.

f Transport of syndecan2 (SDC2) in the Golgi of XYLT2-3xPA+NDST1-9xMyc KI cells after biotin addition observed by SCLIM in 3D. Top two panels: 8 min after addition. Bottom two panels: 15 min after addition.
Some SDC2 signals are localised adjacent to XYLT2 or NDST1 (arrows). Bar, 1 µm.

g Top panel: Time course of the 3D correlation coefficient between SDC2 and XYLT2/NDST1.
Bottom panel: Time course of the correlation coefficient between NDST1 and XYLT2.
The vertical axis shows the correlation coefficient (colocalizing efficiency) between the glycosylation enzymes indicated in the right upper corner. The horizontal axis shows minutes after biotin addition.
The number of cells is 5. For bar plots, values are shown as the mean ± S.E.M.
In a and c-f, images are representative of at least three independent biological replicates.

Supplementary Figure 5

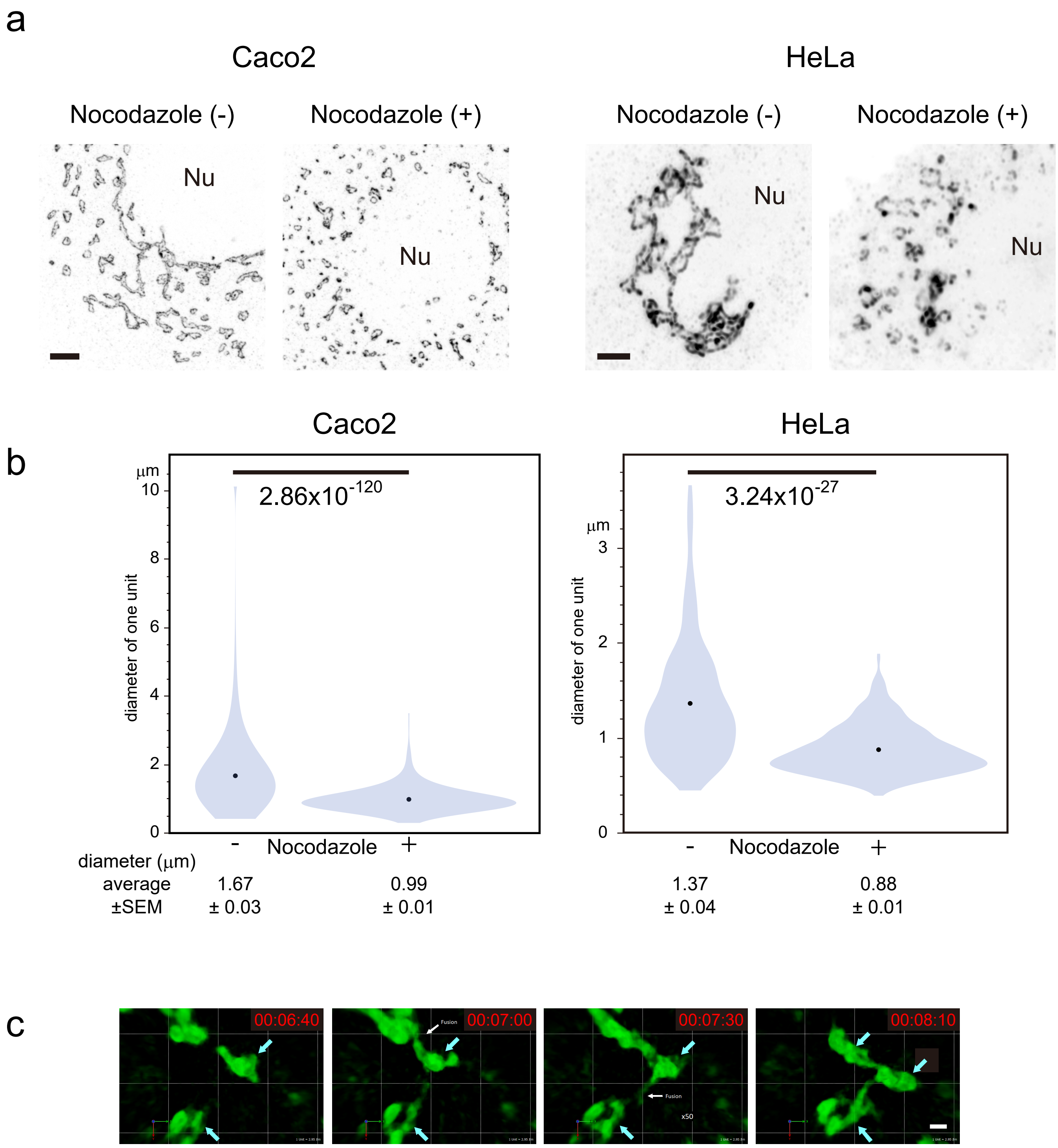

Supplementary Figure 5

a Representative micrographs showing the Golgi units in Caco2 and HeLa cells with and without nocodazole.
The rim of the Golgi unit is stained with giantin and is shown in black. Nu: nucleus. Bars, 5 µm (left), 2 µm (right)

b Distribution of the long diameter of the Golgi units in Caco2 and HeLa cells.
Average diameters ± SEMs are shown below the graph. Statistical significance was determined by two-tailed unpaired t test. P values are depicted in the graph.

c Live imaging of Caco2 cells expressing mNeonGreen-golgin84 imaged by SCLIM in 3D after nocodazole washout (Supplementary movie 3).
Golgi units (blue thick arrows) capture other Golgi units by thin tubules (white thin arrows) and makes a cluster.
Frame rate is 10 seconds/frame (10 seconds/volume). Bar, 1 µm. Images are representative of at least three independent biological replicates.
Imaging modalities and acquisition parameters of SCLIM are described in the materials and methods.

Supplementary Figure 6

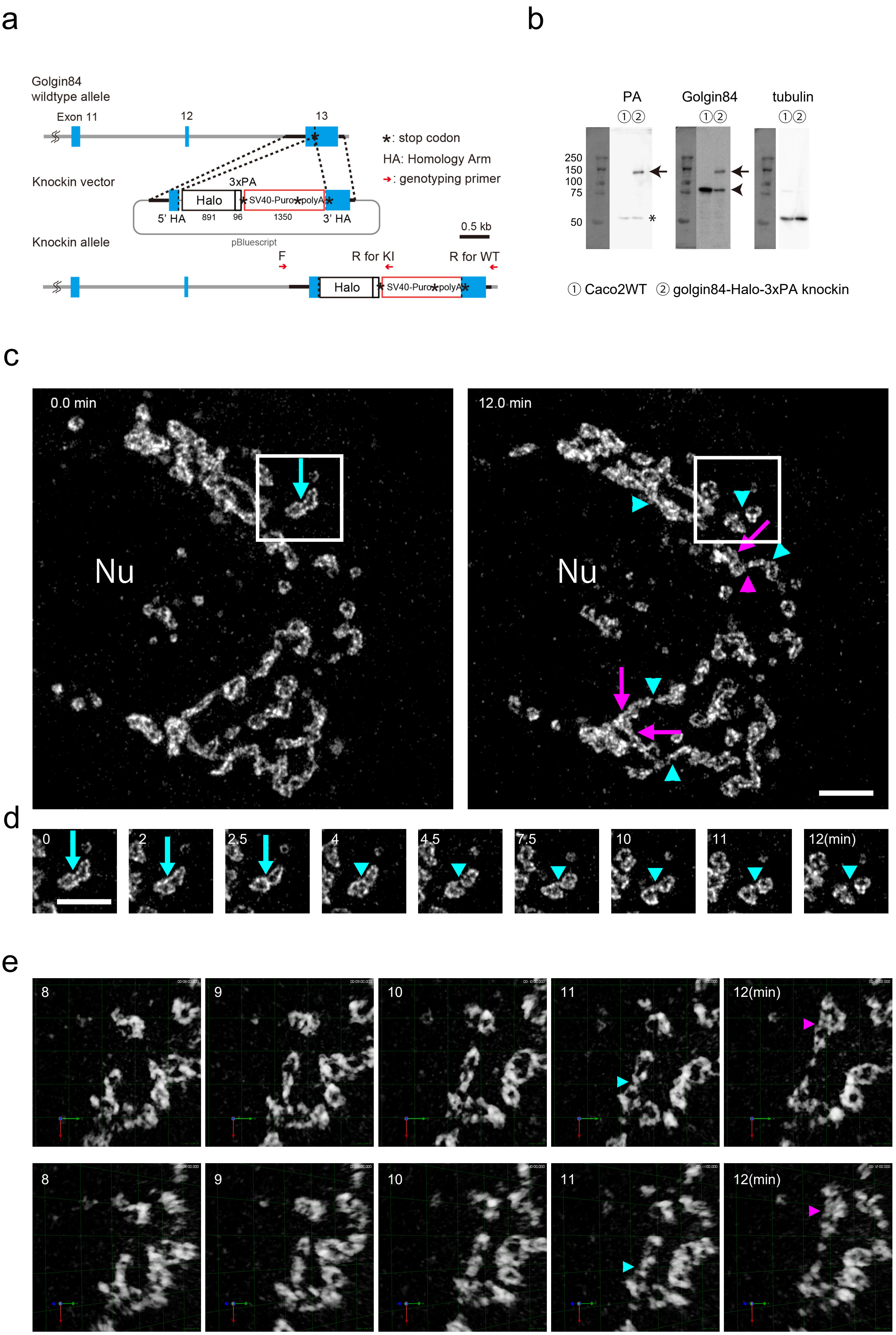

Supplementary Figure 6

a Schematic representation of knockin of tags (Halo-3xPA) into the last coding exon of golgin84.
The positions of primers for genotyping of WT and knockin (KI) alleles are shown as red arrows.

b Western blot of wild-type and golgin84-Halo-3xPA KI Caco2 clones.
Western blot stained with anti-PA (left two lanes), anti-golgin84 (middle two lanes), and anti-tubulin (right two lanes). The membrane used for anti-golgin84 (middle) was reprobed with anti-tubulin (right), and then, finally reprobed with anti-PA antibody to confirm that their molecular weight of golgin84-Halo-3xPA was the same.
An asterisk (*) indicates remaining signals of tubulin. Arrows and an arrowhead indicate a golgin84-Halo-3xPA fusion protein and an endogenous golgin84 protein, respectively.

c, d 2D live imaging of a golgin84-Halo-3xPA KI cell by Airyscan (Supplementary movie 7).

c A micrographs of the Golgi near the nucleus (Nu) at 0 min (left) and 12 min (right).
A region in the small rectangle is showed in d. Blue and magenta arrows indicate separation and fusion between units, respectively. Blue and magenta arrowheads indicate detachment and attachment, respectively.

d A region in the small rectangle in c.
Separation and detachment processes are shown from 0 to 12 min. Bars, 5 µm.

e 3D live imaging of a golgin84-Halo-3xPA KI cell by SCLIM.
Images from 8 to 12 min in Supplementary movie 8 are shown. Detachment and attachment are shown in a blue and a magenta arrowhead, respectively. Images in the bottom row are the images of the top row observed from a different angle. Frame rate is 1 min/frame (1 min/volume). One unit of the grids is 2.94 µm.

Supplementary Figure 7

a

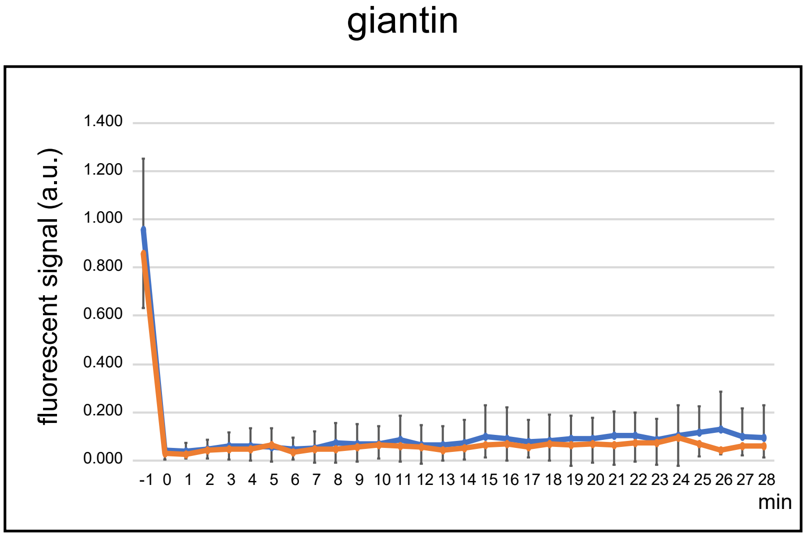

b

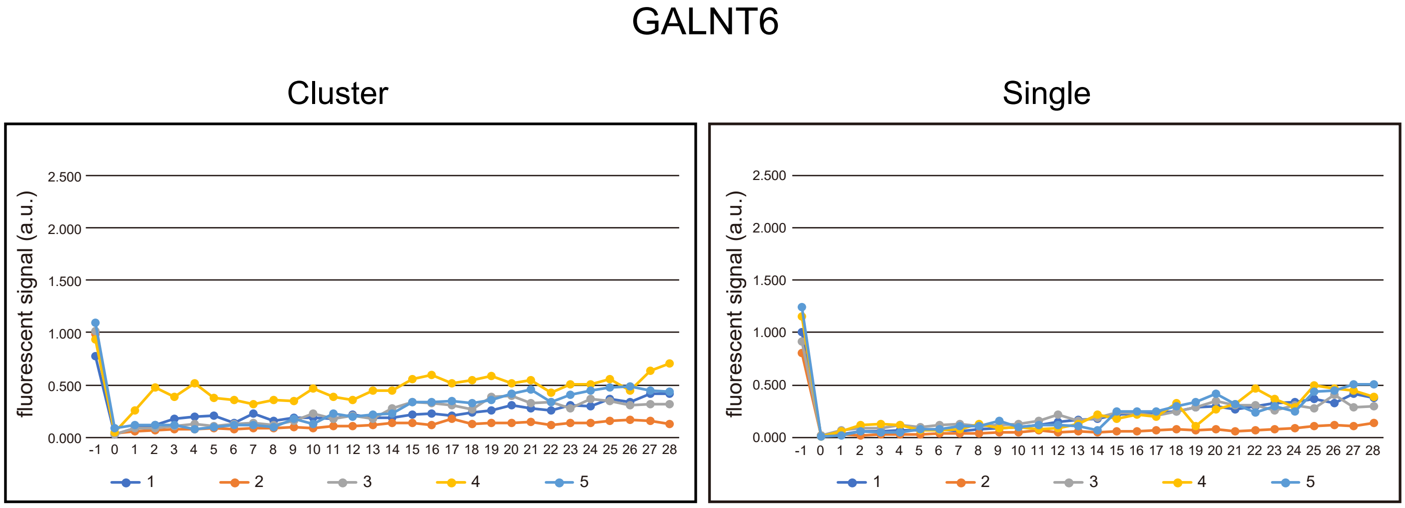

c

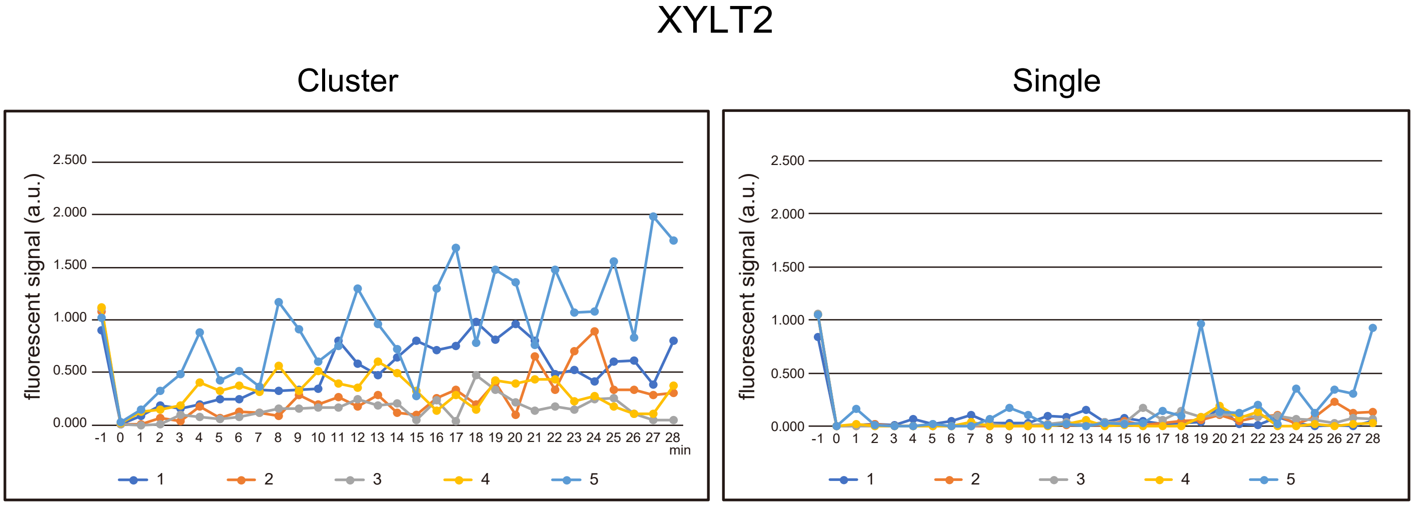

Supplementary Figure 7

Time-dependent FRAP measurements on samples of XYLT2-Halo and GALNT6-Halo KI cells transfected with mNeonGreen giantin.

a Average of giantin signals after photobleaching measured in a cluster (blue line) and an isolated single unit (orange line) from five cells.

b Individual GALNT6-Halo signal from each cell measured in a cluster (left) and an isolated single unit (right).

c Individual XYLT2-Halo signals from each cell measured in a cluster (left) and an isolated single unit (right).

Supplementary Figure 8

a

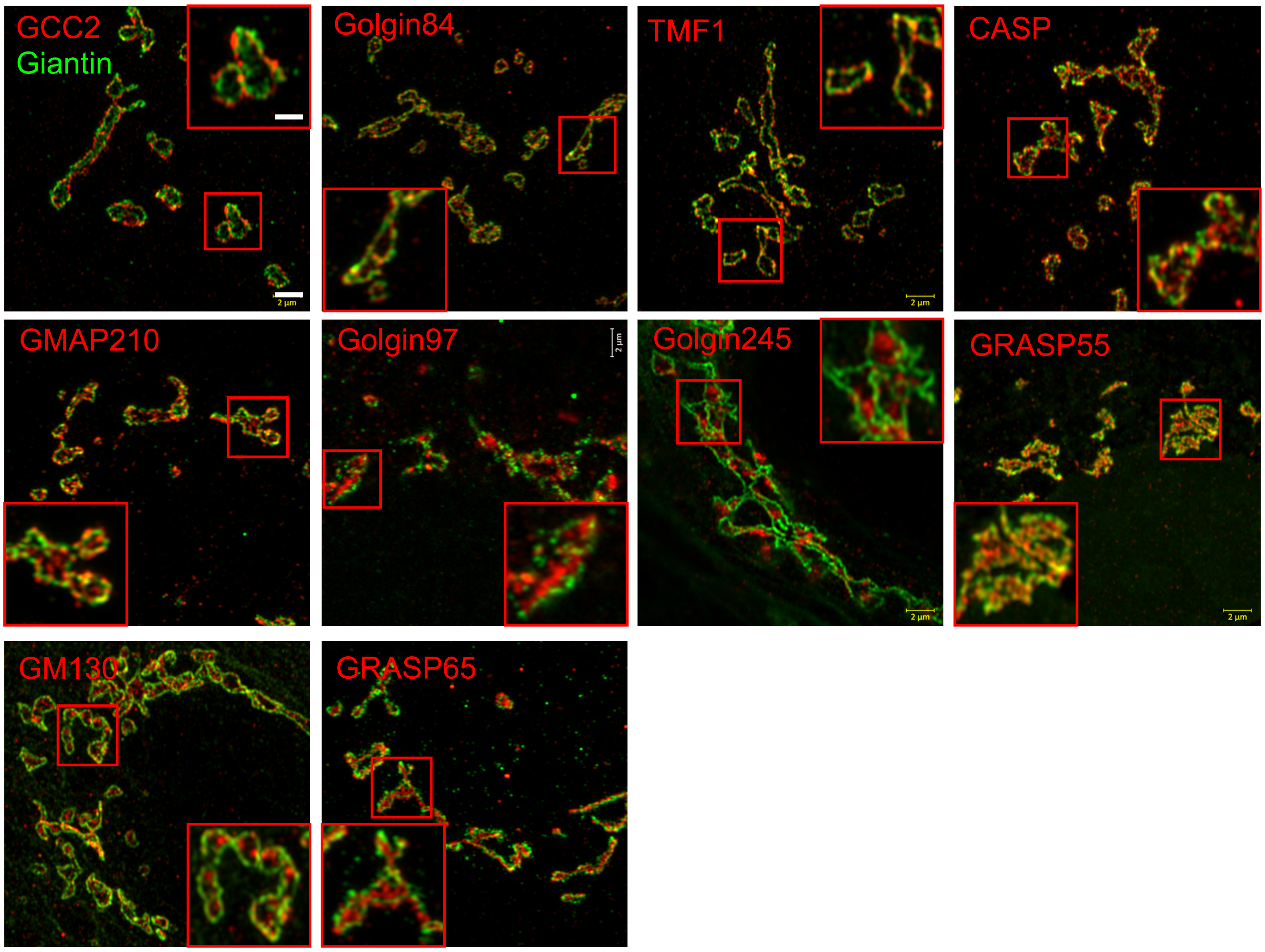

b

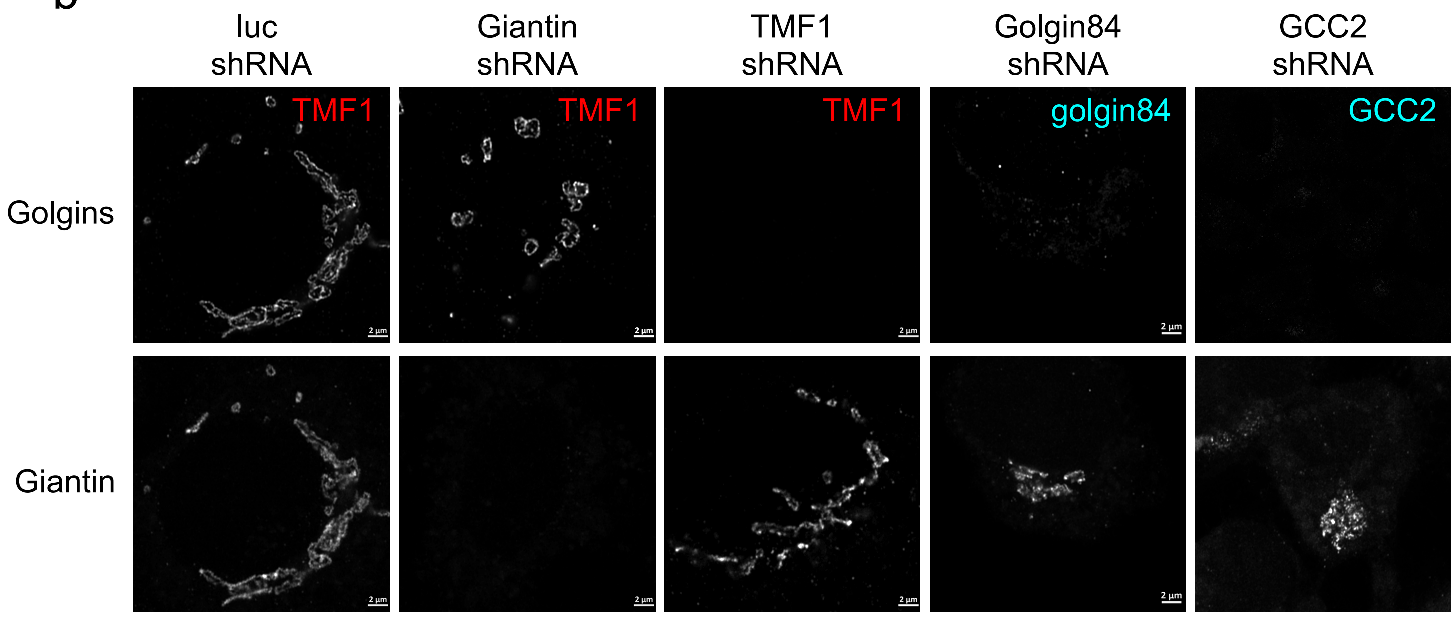

c

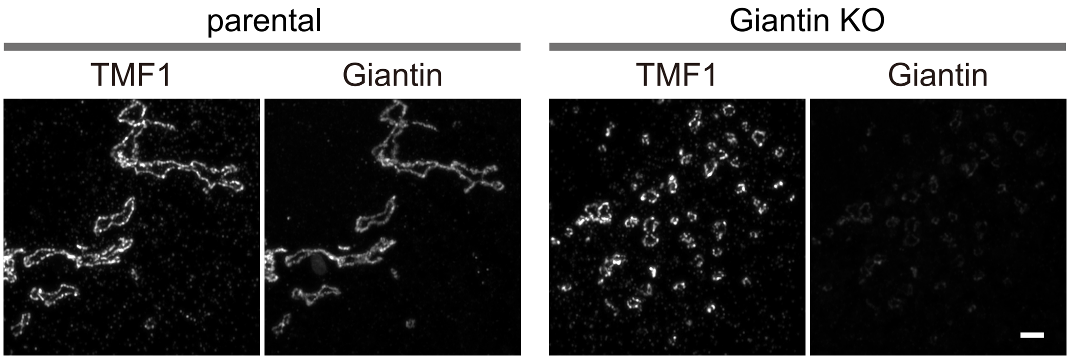

Supplementary Figure 8

a Localisation of golgins and other Golgi-associated proteins relative to giantin in Caco2 cells.
Regions in the rectangles are enlarged as insets. Bars, 2 µm, 1 µm (inset).

b Effects of shRNA of golgins on the distribution of the Golgi units in HeLa cells.
Top panels show the signals of golgins (TMF1, golgin84, and GCC2). The names of stained golgins are indicated on the right upper corner of each panel. Bottom panels show the signals of giantin. Bars, 2 µm.

c Distribution of the Golgi units in a parental and a giantin knockout Caco2 cells.
The rim of the unit is stained by TMF1. Bar, 2 µm.

Supplementary Figure 9

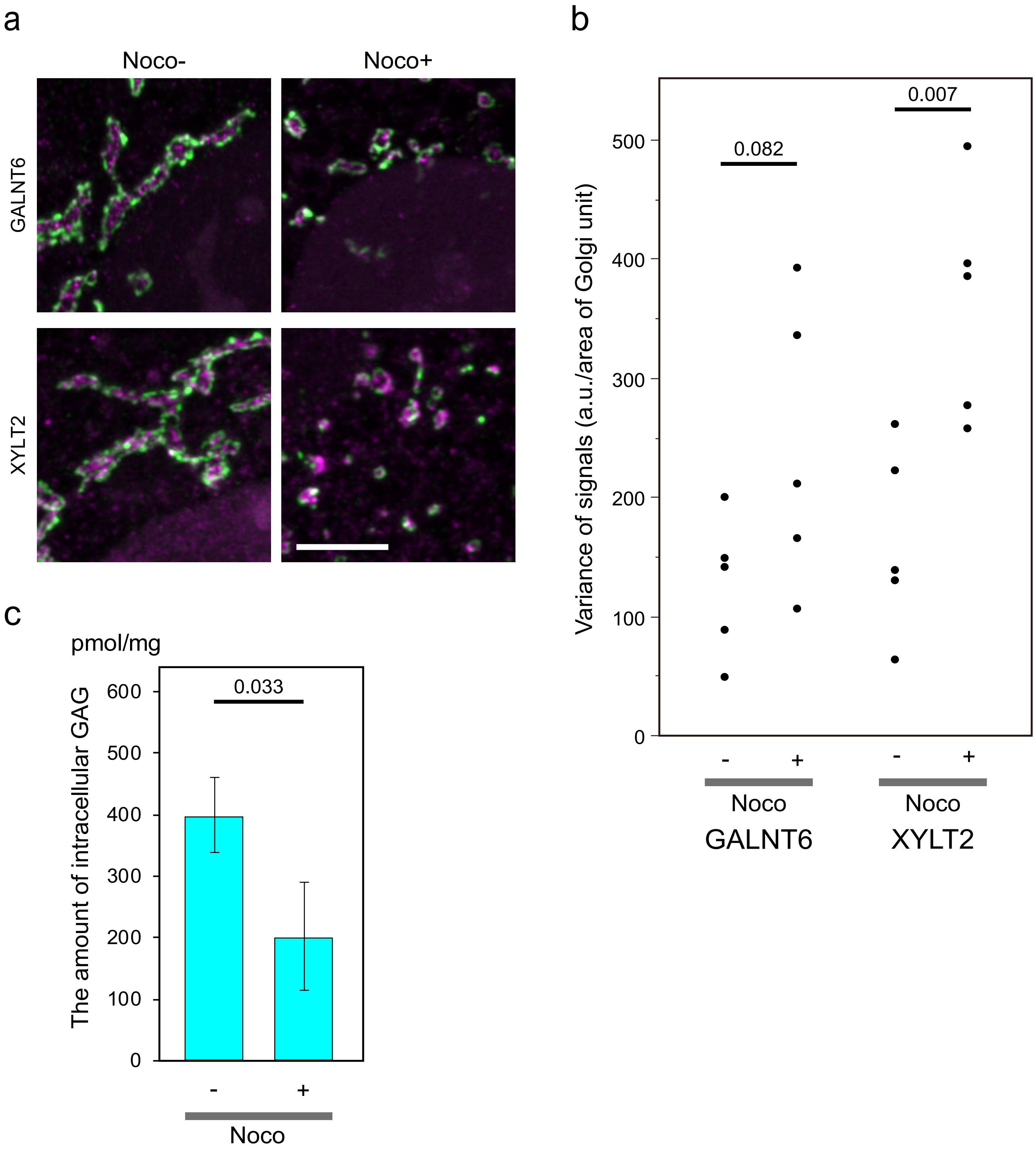

Supplementary Figure 9

a Representative images of nocodazole treated and untreated cells by Airyscan in 2D.

GALNT6 KI (upper panels) and XYLT2 KI (lower panels) Caco2 cells are stained with TMF1 (green) and GALNT6 (magenta) (upper panels) or with TMF1 (green) and XYLT2 (magenta) (lower panels).

Images are representative of at least three independent biological replicates. Bar, 5 µm.

b Effects of giantin knockdown on the distribution of signal intensity of glycosylation enzymes per Golgi unit in Caco2 cells.

The distribution of signal intensity is shown by variance of signals in each cell in Supplementary Fig. 10.

The statistical significance of the variance was determined by the two tailed unpaired t-test.

P values are depicted in the graph.

c Nocodazole treated Caco2 cells display reduced amount of GAG (heparan sulphate).

The statistical significance was determined by the two tailed unpaired t-test.

P values are depicted above the plots in the graph.

Supplementary Figure 10

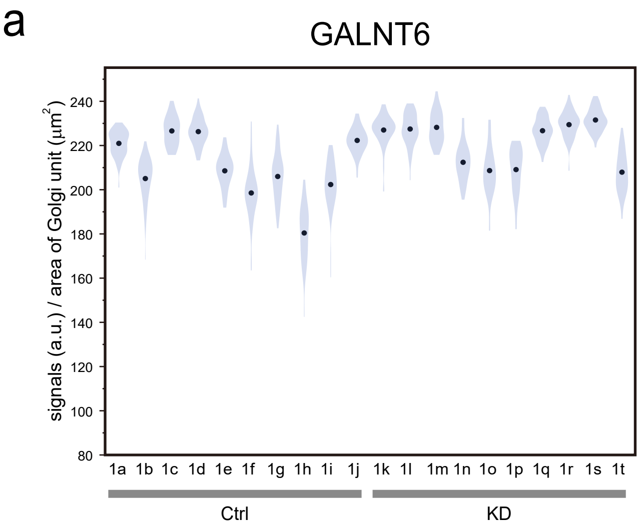

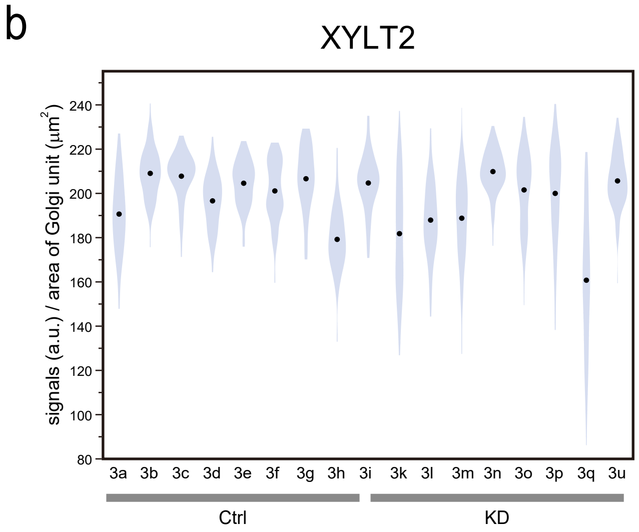

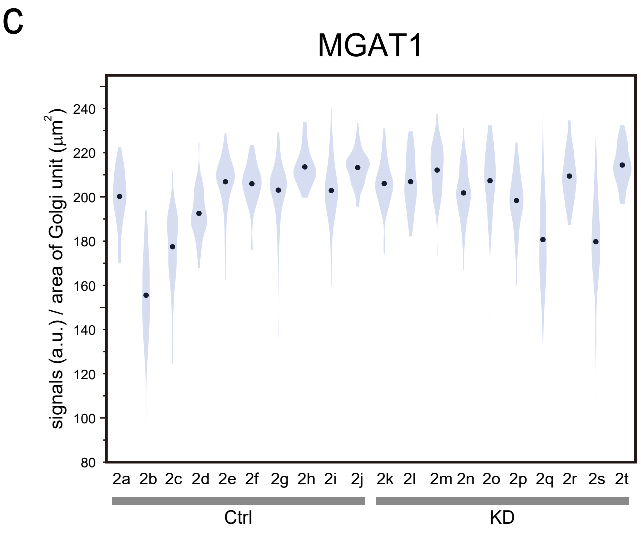

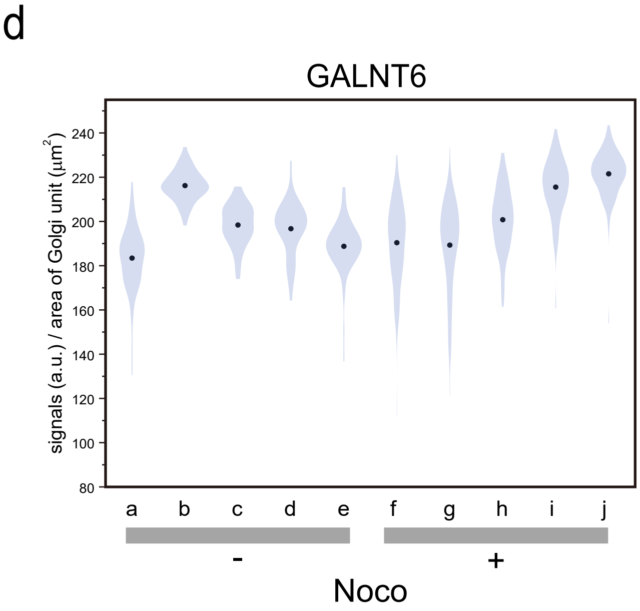

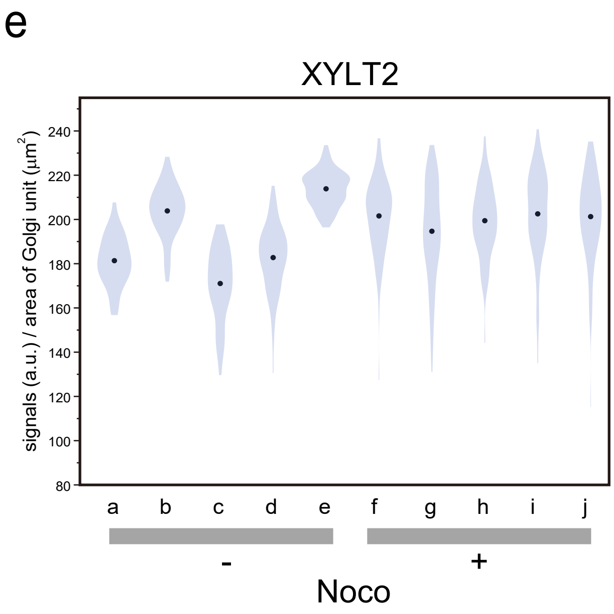

Supplementary Figure 10

a-c Effects of giantin knockdown on the distribution of signal intensity of glycosylation enzymes per Golgi unit in Caco2 cells. **a**: GALNT6, **b**: XYLT2, **c**: MGAT1.

The distribution of signal intensity is shown by a violin plot. The number of cells is 10 (knockdown) and 10 (control).

d, e Effects of nocodazole on the distribution of signal intensity of glycosylation enzymes per Golgi unit in Caco2 cells. **d**: GALNT6, **e**: XYLT2.

The distribution of signal intensity is shown by a violin plot. The number of cells is 5 (knockdown) and 5 (control).

Supplementary Figure 11

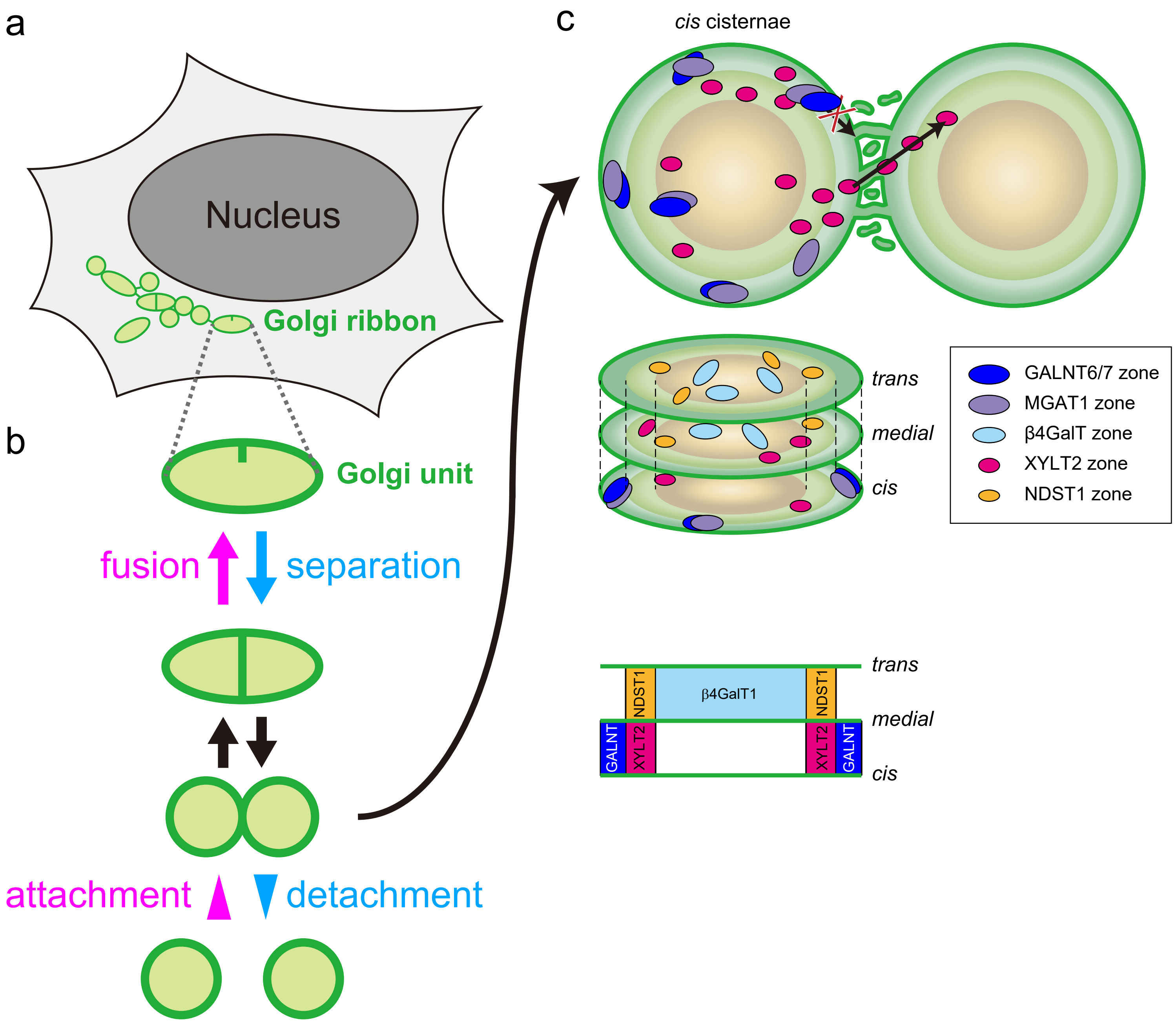

Supplementary Figure 11

Cartoon illustration showing the structure of the Golgi complex.

a Mammalian Golgi is composed of a number of Golgi units and tubules between them.

b Magnified view of a Golgi unit. Golgi units undergo dynamic morphological changes.
One Golgi unit develops a partition and separates a unit into two (blue arrow). Separated units frequently detach (blue arrowhead). This reaction also occurs in the reverse direction (magenta arrow and arrowhead).

c Localisation and movement of the zones within and between the Golgi units.
(top) A hypothesis to explain the interunit transport. XYLT2 zones (red) are small enough to pass through the narrow channel between two units. However, GALNT6/7 zones (dark blue) are too large to pass through.
(middle) GALNT6/7 zones and MGAT1 zones (gray) are colocalised near the rim of the Golgi units.
(bottom) A side view of a Golgi unit and the area of cisternae in which zones are mainly localised.
The cisternae between trans and medial cisternae and those between medial and cis cisternae are omitted.

Supplementary Table 1

Measurement of fusion and separation events of the Golgi units in live imaging samples #1-6 transfected with mNeon Green-giantin or mNeon Green-golgin84 taken by Airyscan

	**Area of the Golgi [μm^2]**								
sample #	start	end	**average**	min	Transfected cDNA	fusion	separation	fusion/h/μm^2	separation/h/μm^2
1	61.962	70.513	66.2375	60	giantin	7	5	0.105680317	0.075485941
2	58.281	50.521	54.401	60	giantin	9	6	0.165438135	0.11029209
3	52.53	40.778	46.654	30	giantin	4	6	0.171475115	0.257212672
4	22.554	21.72	22.137	30	giantin	9	8	0.813118309	0.72277183
5	52.905	52.025	52.465	30	giantin	6	6	0.228723911	0.228723911
6	38.57	33.323	35.9465	60	golgin84	6	10	0.16691472	0.278191201

Supplementary Table 2

Vectors and tags used for generating knockin cells.

backbone vector	tag	source of tag	Drug resistance	inserted site of pBS	usable restriction enzyme site for insertion of 5' HA	usable restriction enzyme site for insertion of 3' HA
pBS-9xMyc-puro-loxP #1	9xMyc	pMK76	Puromycin	PstI	SacI, NotI, SpeI	EcoRI, EcoRV, SalI, XhoI, ApaI
pBS-9xMyc-puro-loxP #2					ApaI, XhoI, SalI, EcoRV, EcoRI	SpeI, NotI, SacI
pBS-3xPA-Hygro-loxP #1	3xPA	pCD-PAx3	Hygromycin B	BamHI	SacI, NotI, XbaI, SpeI	EcoRV, SalI, XhoI, ApaI
pBS-3xPA-Hygro-loxP #2					ApaI, XhoI, SalI, EcoRV	SpeI, XbaI, NotI, SacI
pBS-Halo-3xPA-Puro #1	Halo-3xPA	pHaloTag-EGFP (addgene #86629)	Puromycin	PstI	SacI, NotI, XbaI, SpeI	EcoRI, EcoRV, ClaI, ApaI
pBS-Halo-3xPA-Puro #2					ApaI, ClaI, EcoRV, EcoRI	SpeI, XbaI, NotI, SacI
pBS-2xSNAP-9xMyc-Hygro #1	2xSNAP-9xMyc	pME-puro-SNAP-FLAG-CD59 (addgene #50374)	Hygromycin B	BamHI	SacI, NotI, SpeI	EcoRV, SalI, XhoI, ApaI
pBS-2xSNAP-9xMyc-Hygro #2					ApaI, XhoI, SalI, EcoRV	SpeI, NotI, SacI
pBS-3xV5-Blast #1	3xV5	-	Blasticidin S	PstI	SacI, SacII, NotI, XbaI	EcoRI, EcoRV, ClaI, SalI
pBS-3xV5-Blast #2					SalI, ClaI, EcoRV, EcoRI	XbaI, NotI, SacII, SacI
