# Supplementary material for: Dynamic movement of the Golgi unit and its glycosylation enzyme zones
Source: Nat Commun. 2024 May 27;15:4514. doi: 10.1038/s41467-024-48901-1 (PMC11130159; doi:10.1038/s41467-024-48901-1)
Supplement: Supplementary file 3 — Description of Additional Supplementary Files [file 41467_2024_48901_MOESM3_ESM.pdf]

Description of Additional Supplementary Files

File Name: Supplementary movie 1

The 3D architecture of the Golgi ribbon in a fixed Caco2 cell observed by SCLIM. There is one Golgi unit at the center of the movie ('Unit 1') connected with the other unit ('Unit 2') by a giantin-positive tubule (a vertical arrow which appears first). Unit 2 has two additional tubules which connects other units on the left (a vertical arrow which appears next) and the bottom (a horizontal arrow).

File Name: Supplementary movie 2

3D time-lapse observation of a Caco2 cell expressing mNeonGreen-golgin84 by SCLIM after addition of nocodazole. Arrows indicate gaps between the units or tubules after treatment. One segment of the grids is 1.81mm. 15x real time.

File Name: Supplementary movie 3

3D time-lapse observation of a Caco2 cell expressing mNeonGreen-golgin84 by SCLIM after washout of nocodazole. Thin white arrows indicate fusion mediated by tubules. Thick blue arrows indicate Golgi units. One segment of the grids is 2.95mm. 20x or 100x real time.

File Name: Supplementary movie 4

2D time-lapse observation of a Caco2 cell expressing mNeonGreen-giantin, which was obtained by Airyscan confocal microscopy. The blue and magenta arrows indicate separation and fusion of the Golgi units, respectively. The blue and magenta arrowheads indicate detachment and attachment of the Golgi units, respectively. The scale bar is 5 µm. 300 x real time.

File Name: Supplementary movie 5

3D time-lapse observation of a Caco2 cell expressing mNeonGreen-golgin84 by SCLIM. Tilted arrows indicate a partition between units during their fusion. Vertical arrows indicate an elongating tubule from the unit and the point of scission. 15 x real time. One segment of the grids is 2.98 µm.

File Name: Supplementary movie 6

2D time-lapse observation of a T47D cell expressing mNeonGreen-giantin using Airyscan confocal microscopy. In the former part of the movie, magenta arrows indicate

fusion of the Golgi units. Blue and magenta arrowheads indicate detachment and attachment of the Golgi units, respectively. In the latter part, blue arrows indicate separation and detachment because these two steps cannot be separated in this movie. The scale bar is 2 μm. 300 x real time.

File Name: Supplementary movie 7
2D Time-lapse observation of a Golgin 84-Halo knockin (KI) Caco2 cell using Airyscan. The blue and magenta arrows indicate separation and fusion of the Golgi units, respectively. The blue and magenta arrowheads indicate detachment and attachment of the Golgi units, respectively. The scale bar is 5 μm. 150 x real time.

File Name: Supplementary movie 8
3D time-lapse observation of a golgin 84-Halo KI Caco2 cell by SCLIM. The blue and magenta arrows indicate separation and fusion of the Golgi units, respectively. The blue and magenta arrowheads indicate detachment and attachment of the Golgi units, respectively. The scale bar is 2.94 μm. 300 x real time.

File Name: Supplementary movie 9
3D time-lapse observation of a GALNT6-Halo KI Caco2 cell expressing mScarlet-golgin84 by SCLIM. GALNT6 and giantin are shown in magenta and green, respectively. A small rectangle at the beginning of the movie is the same region as shown in Fig. 6a. A small rectangle at the latter half of the movie shows the movement of GALNT6 at the similar level to giantin in a small unit . Blue and magenta arrowheads indicate detachment and attachment of the Golgi units, respectively. One segment of the grids is 2.98 μm. 30 x real time.

File Name: Supplementary movie 10
3D time-lapse observation of an XYLT2-Halo KI Caco2 cell expressing mNeonGreen-golgin84 by SCLIM. XYLT2 and giantin are shown in magenta and green, respectively. A small rectangle is the same region as shown in Fig. 6b. The blue and magenta arrowheads indicate detachment and attachment of the Golgi units, respectively, which is followed by fusion shown by an arrow. At the end of the movie, the movement of XYLT2 at the similar level to giantin is shown by white arrows. Finally, the arrowhead on the right shows transport of small units or fragments that fuse to other units. One segment of the grids is 1.51 μm. 15 x real time.

File Name: Supplementary movie 11
3D time-lapse observation of an NDST1-Halo KI Caco2 cell expressing mNeonGreen-golgin84 by SCLIM. NDST1 and giantin are shown in magenta and green, respectively. One segment of the grids is 2.92 μm. 15 x real time.

File Name: Supplementary movie 12
3D time-lapse observation of a GALNT6-Halo KI T47D cell expressing mNeonGreen-giantin by SCLIM. GALNT6 and giantin are shown in magenta and green, respectively. In the latter part of the movie, the movement of XYLT2 at the similar level to giantin is shown by the side view of an enlarged unit. One segment of the grids is 3.01 μm. 15 x real time.

File Name: Supplementary movie 13
3D time-lapse observation of an XYLT2-Halo+GALNT6-SNAPx2 double KI Caco2 cell by SCLIM. XYLT2 and GALNT6 are shown in magenta and green, respectively. A small rectangle is the same region as shown in Fig. 6c. One segment of the grids is 1.09 μm. 15 x real time.

File Name: Supplementary movie 14
2D time-lapse observation after photobleaching of a GALNT6-Halo KI Caco2 cell expressing mNeonGreen-giantin using Airyscan. A green rectangle is the target of photobleaching. GALNT6 and giantin are shown in magenta and green, respectively. Arrowheads: A bleached single unit, Horizontal arrows: A bleached unit in cluster. Vertical arrows: a unit in the nonbleached region. The scale bar is 2 μm. 300 x real time.

File Name: Supplementary movie 15
2D time-lapse observation after photobleaching of a XYLT2-Halo KI Caco2 cell expressing mNeonGreen-giantin using Airyscan. A blue rectangle is the target of photobleaching. XYLT2 and giantin are shown in magenta and green, respectively. Arrowheads: A bleached single unit, Horizontal arrows: A bleached unit in cluster. Vertical arrows: a unit in the nonbleached region. The scale bar is 2 μm. 300 x real time.
