# Supplementary material for: Dynamic movement of the Golgi unit and its glycosylation enzyme zones
Source: Nat Commun. 2024 May 27;15:4514. doi: 10.1038/s41467-024-48901-1 (PMC11130159; doi:10.1038/s41467-024-48901-1)
Supplement: Supplementary file 19 — Reporting Summary [file 41467_2024_48901_MOESM19_ESM.pdf]

nature portfolio

Corresponding author(s): *Double-anonymous peer review submissions: write DAPR and your manuscript number here instead of author names.*

Last updated by author(s): *YYYY-MM-DD*

Reporting Summary

Nature Portfolio wishes to improve the reproducibility of the work that we publish. This form provides structure for consistency and transparency in reporting. For further information on Nature Portfolio policies, see our Editorial Policies and the Editorial Policy Checklist.

Statistics

For all statistical analyses, confirm that the following items are present in the figure legend, table legend, main text, or Methods section.

n/a	Confirmed	
☐	☒	The exact sample size (n) for each experimental group/condition, given as a discrete number and unit of measurement
☐	☒	A statement on whether measurements were taken from distinct samples or whether the same sample was measured repeatedly
☐	☒	The statistical test(s) used AND whether they are one- or two-sided<br>*Only common tests should be described solely by name; describe more complex techniques in the Methods section.*
☒	☐	A description of all covariates tested
☐	☒	A description of any assumptions or corrections, such as tests of normality and adjustment for multiple comparisons
☐	☒	A full description of the statistical parameters including central tendency (e.g. means) or other basic estimates (e.g. regression coefficient) AND variation (e.g. standard deviation) or associated estimates of uncertainty (e.g. confidence intervals)
☐	☒	For null hypothesis testing, the test statistic (e.g. F, t, r) with confidence intervals, effect sizes, degrees of freedom and P value noted<br>*Give P values as exact values whenever suitable.*
☒	☐	For Bayesian analysis, information on the choice of priors and Markov chain Monte Carlo settings
☒	☐	For hierarchical and complex designs, identification of the appropriate level for tests and full reporting of outcomes
☐	☒	Estimates of effect sizes (e.g. Cohen's d, Pearson's r), indicating how they were calculated

Our web collection on statistics for biologists contains articles on many of the points above.

Software and code

Policy information about availability of computer code

Data collection	ZEN software (Carl Zeiss) for Airyscan. custom-made software (Yokogawa Electric) for SCLIM. NIS-Elements imaging software (Nikon Instruments Inc.) for STORM.
Data analysis	Volocity, Image J

For manuscripts utilizing custom algorithms or software that are central to the research but not yet described in published literature, software must be made available to editors and reviewers. We strongly encourage code deposition in a community repository (e.g. GitHub). See the Nature Portfolio guidelines for submitting code & software for further information.

Data

Policy information about availability of data

All manuscripts must include a data availability statement. This statement should provide the following information, where applicable:
- Accession codes, unique identifiers, or web links for publicly available datasets
- A description of any restrictions on data availability
- For clinical datasets or third party data, please ensure that the statement adheres to our policy

The authors declare that all data supporting the findings of this study are available within the article and its supplementary information or are available from the corresponding authors on request. Source data are provided with this paper.

Research involving human participants, their data, or biological material

Policy information about studies with human participants or human data. See also policy information about sex, gender (identity/presentation), and sexual orientation and race, ethnicity and racism.

Reporting on sex and gender	This information has not been collected.
Reporting on race, ethnicity, or other socially relevant groupings	This information has not been collected.
Population characteristics	This information has not been collected.
Recruitment	This information has not been collected.
Ethics oversight	This information has not been collected.

Note that full information on the approval of the study protocol must also be provided in the manuscript.

Field-specific reporting

Please select the one below that is the best fit for your research. If you are not sure, read the appropriate sections before making your selection.

[x] Life sciences [ ] Behavioural & social sciences [ ] Ecological, evolutionary & environmental sciences

For a reference copy of the document with all sections, see nature.com/documents/nr-reporting-summary-flat.pdf

Life sciences study design

All studies must disclose on these points even when the disclosure is negative.

Sample size	In case of imaging, values are consistent enough in the same set of experiment. Thus, usually we can see statistically significance by comparing the sample more than five.
Data exclusions	No data were excluded.
Replication	To verify the reproducibility, we simply repeated taking pictures of images of more than five samples and/or on different occasions. For quantification of proteoglycan, we used different shRNA for knockdown.
Randomization	In our case, we selected cells well- stained by antibodies or ligands. We were not able to predict the outcome, such as correlation coefficient, from the pictures at the time of sample collection. Therefore, we did not need to randomize samples.
Blinding	As described above , the outcome cannot be predicted when we collect the data. Therefore, we did not need blinding.

Reporting for specific materials, systems and methods

We require information from authors about some types of materials, experimental systems and methods used in many studies. Here, indicate whether each material, system or method listed is relevant to your study. If you are not sure if a list item applies to your research, read the appropriate section before selecting a response.

Materials & experimental systems

n/a	Involved in the study	
[ ]	[x]	Antibodies
[ ]	[x]	Eukaryotic cell lines
[x]	[ ]	Palaeontology and archaeology
[x]	[ ]	Animals and other organisms
[x]	[ ]	Clinical data
[x]	[ ]	Dual use research of concern
[x]	[ ]	Plants

Methods

n/a	Involved in the study	
[x]	[ ]	ChIP-seq
[x]	[ ]	Flow cytometry
[x]	[ ]	MRI-based neuroimaging

Antibodies

Antibodies used	anti-PA rat monoclonal antibody (Fuji Film), anti-Myc rabbit polyclonal antibody (MBL Life Science #562), anti-V5 mouse monoclonal antibody (ThermoFisher #R960-25), anti-GALNT6 mouse monoclonal antibody and rabbit polyclonal antibody (ref 51), anti-β4GalT1 mouse monoclonal antibody (ref 19), anti-C1GalT1 mouse monoclonal antibody (Millipore #MABT1555), anti-Giantin (N terminal) mouse monoclonal antibody (Abcam #ab37266), anti-Giantin (C terminal) rabbit polyclonal antibody (BioLegend #924301), anti-TMF1

rabbit polyclonal antibody (Sigma #HPA008729), anti-golgin84 rabbit polyclonal antibody (Sigma #HPA000992), anti-GCC2 rabbit polyclonal antibody (Sigma #HPA035849), anti-CASP rabbit monoclonal antibody (Abcam #ab182216), anti-GMAP210 rabbit polyclonal antibody (Proteintech #26456-1-AP), anti-golgin97 polyclonal antibody (ref 52), anti-golgin245 mouse monoclonal antibody (BD Biosciences # 611281), anti-GRASP55 rabbit polyclonal antibody (Novus Biologicals #NBP2-38244), anti GRASP65 polyclonal antibody (ref 53), anti-GM130 mouse monoclonal antibody (BD Biosciences #610822), anti-DYKDDDDK mouse monoclonal antibody (clone #1E6, Fuji Film). GALNT7 rabbit polyclonal antibody was raised in rabbits by injecting peptide encoding 40-56 amino acid residues of human GALNT7. The following secondary antibodies and reagents were used: donkey anti-mouse IgG (H+L) highly cross-adsorbed secondary antibody, Alexa Fluor 488 (Invitrogen, #A-21202), donkey anti-rabbit IgG (H+L) highly cross-adsorbed secondary antibody, Alexa Fluor 568 (Invitrogen, #A-10042), donkey anti-rat IgG (H+L) highly cross-adsorbed secondary antibody, Cy5 (Jackson Immuno Research, #712-175-153), HRP-labelled donkey anti-rat and anti-rabbit antibodies (Jackson ImmunoResearch Laboratories), HRP-conjugated streptavidin (Jackson ImmunoResearch Laboratories) goat anti-rabbit or anti-rat Alexa Fluor 488-labelled FluoroNanogold (Nanoprobes), biotin-labelled anti-mouse or anti-rabbit (Vectastain ABC kit Elite from Vector Laboratories)

Validation

Validation was based on the data sheet of manufacturers and researchers. If necessary, additional validation was performed largely by Western blot and/or immunofluorescence.

Eukaryotic cell lines

Policy information about cell lines and Sex and Gender in Research

Cell line source(s)

We used Caco2, T47D, and HeLa cells obtained from ATCC.

Authentication

These cell lines were authentified by ATCC.

Mycoplasma contamination

They were not tested for mycoplasma contamination in our hands.

Commonly misidentified lines
(See ICLAC register)

Name any commonly misidentified cell lines used in the study and provide a rationale for their use.

Plants

Seed stocks

Report on the source of all seed stocks or other plant material used. If applicable, state the seed stock centre and catalogue number. If plant specimens were collected from the field, describe the collection location, date and sampling procedures.

Novel plant genotypes

Describe the methods by which all novel plant genotypes were produced. This includes those generated by transgenic approaches, gene editing, chemical/radiation-based mutagenesis and hybridization. For transgenic lines, describe the transformation method, the number of independent lines analyzed and the generation upon which experiments were performed. For gene-edited lines, describe the editor used, the endogenous sequence targeted for editing, the targeting guide RNA sequence (if applicable) and how the editor was applied.

Authentication

Describe any authentication procedures for each seed stock used or novel genotype generated. Describe any experiments used to assess the effect of a mutation and, where applicable, how potential secondary effects (e.g. second site T-DNA insertions, mosiacism, off-target gene editing) were examined.
